# Impact of an in-situ Cr(VI)-contaminated site remediation on the groundwater

**DOI:** 10.1007/s11356-019-07513-9

**Published:** 2020-01-14

**Authors:** Klaus Philipp Sedlazeck, Daniel Vollprecht, Peter Müller, Robert Mischitz, Reto Gieré

**Affiliations:** 1 Montanuniversitaet Leoben, Chair of Waste Processing Technology and Waste Management, Franz-Josef-Straße 18, 8700 Leoben, Austria; 2 ferroDECONT GmbH, Peter-Tunner-Straße 19, 8700 Leoben, Austria; 3 Department of Earth and Environmental Science, University of Pennsylvania, 240 South 33rd Street, Philadelphia, PA 19104-6316 USA

**Keywords:** Cr(VI) remediation, In-situ treatment, Sodium dithionite injection, Cr(VI) mobilization, Cr(VI) reduction, Rebound effect

## Abstract

This study presents the latest results of the groundwater monitoring of a research project, which tested an innovative pump and treat method in combination with an in-situ remediation. This technique was assessed on an abandoned site in Austria, where two hot spots of hexavalent chromium (Cr(VI)) were located. For the in-situ remediation, a strong reducing agent (sodium dithionite) was injected into the underground to reduce Cr(VI) to Cr(III) by using different injection strategies. Throughout this treatment, part of the Cr(VI) is mobilized and not instantly reduced. To prevent a further spreading of the mobilized Cr(VI), the pump and treat method, which uses zero-valent iron to clean the groundwater, was installed downgradient of the hot spots. Based on the groundwater sample analyses, it was possible to distinguish different remediation phases, characterized by excess chromate and excess sulfite. During the excess sulfite conditions, Cr(VI) was successfully removed from the system, but after terminating the sodium dithionite injection, the Cr(VI) rebounded.

**Electronic supplementary material:**

The online version of this article (10.1007/s11356-019-07513-9) contains supplementary material, which is available to authorized users.

## Introduction

A recent review of current contaminated-site statistics has identified a total of 342,000 such sites in Europe, and estimated an even higher number (2.5 million) of potentially contaminated sites (Panagos et al. 2013). These data highlight the essential importance of continuing to pursue research in this field. In Austria, the declared national remediation target is a clean-up of nearly all heavily contaminated sites by 2050, and it is assumed that a total clean-up would require another 100 years of remediation work, by using today’s state-of-the-art technology (Skala et al. 2007).

Each contaminated-site remediation project challenges the appropriate authorities, because no two sites resemble each other. Not only the huge variety of contaminants but also different types of land use influence the choice of the most suitable remediation method for a particular site, especially those located in developed areas. Restrictions from existing development, financial reasons, and operational aspects force researchers to continuously improve and/or adapt remediation and safeguarding techniques.

Consequently, the Montanuniversitaet Leoben invented an innovative technique for the decontamination of heavy metal–contaminated waters, and it was tested during the course of several research projects. This new technique utilizes zero-valent iron (Fe(0)) in a fluidized bed reactor for groundwater remediation in combination with an in-situ soil remediation technique (Müller et al. 2014), where the carcinogenic, toxic, highly mobile hexavalent form of chromium (Cr(VI)) is reduced to the non-toxic, immobile trivalent form (Cr(III)) (Lilli et al. 2015; Rajapaksha et al. 2018; Li et al. 2019). A detailed description of the development of this remediation method is provided by Müller et al. (2014).

Our paper presents the results of the latest research project that uses this new approach for a hot spot remediation, which was tested on a chromium (Cr)-contaminated site in Carinthia, Austria.

## Materials and methods

### Site description and preceding work

The following description of the studied site is summarized from the contaminated-site registry of the Environment Agency Austria (EAA) (Umweltbundesamt 2013). On the studied site, a leather tannery was operated from 1922 to 2017, covering an area of 30,000 m^2^. About 15,000 m^2^ of this area are declared contaminated. It is located north-east of downtown Klagenfurt, the capital of Carinthia in the southern part of Austria, about 230 km southwest of Vienna. Today, leather production has ceased, but the infrastructure is maintained to some extent (Müller et al. 2007, 2014; Sedlazeck et al. 2017).

**Fig. 1:**
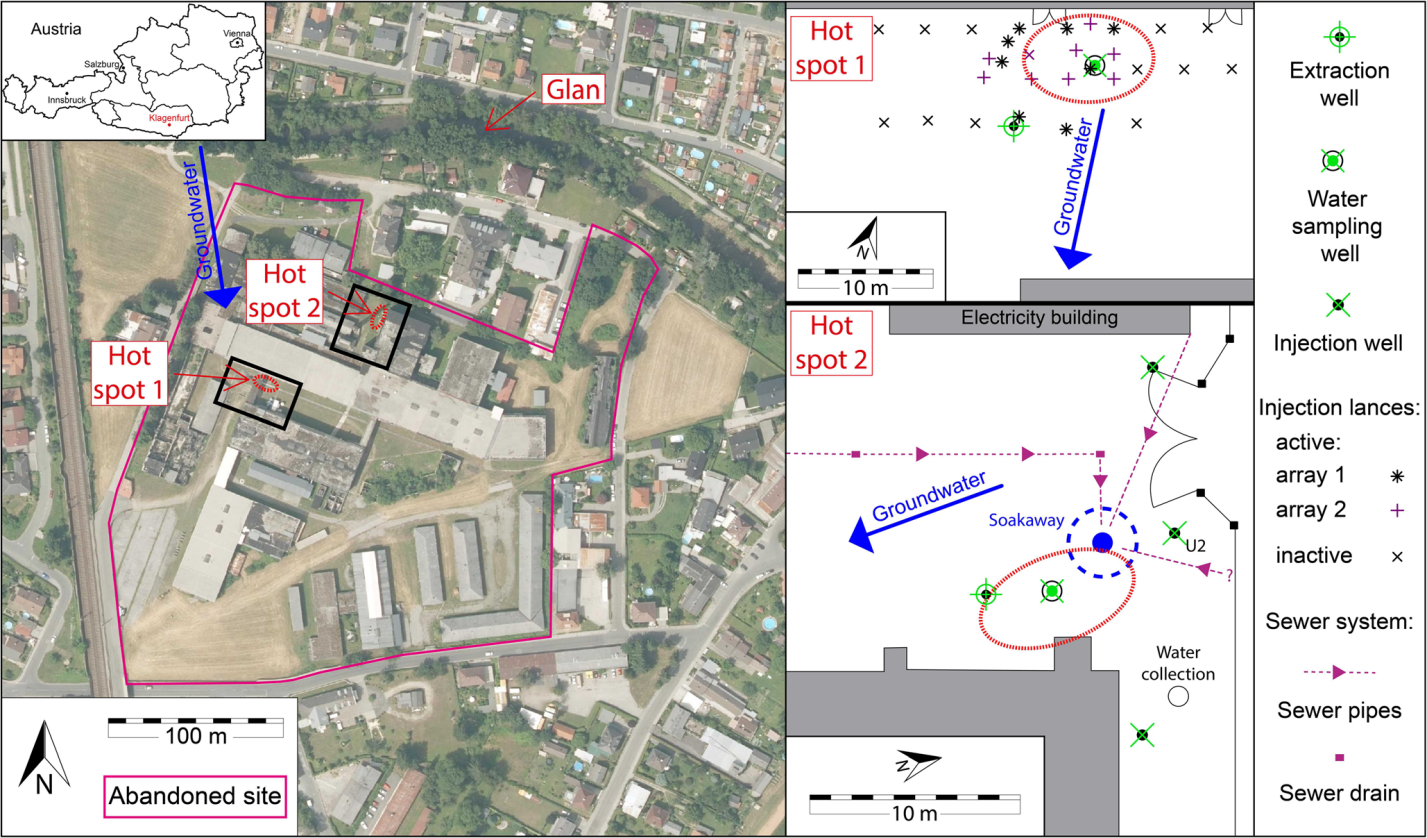
Left: Satellite image of the area northeast of Klagenfurt, showing the studied abandoned site (border represented by magenta line), the identified hot spots, the river Glan, and the direction of the groundwater flow (modified from Kagis (2017) and Sedlazeck et al. (2017)). Right: Close-up schemes of the two identified hot spots, marking the locations of the groundwater extraction wells, water-sampling wells, injection wells and lances, and the groundwater flow directions. Also marked at hot spot 2 (bottom) is the mapped sewer system and the identified contamination source, the soakaway, from where the contaminant infiltrated the aquifer (modified from Müller et al. (2007) and Sedlazeck et al. (2017))

The contaminated site is located at the northern rim of the Klagenfurt basin, where the subsurface consists of Neogene and Quaternary alluvial sediment sequences, deposited by the river Glan (Nemes et al. 1997; Heberer et al. 2017). The surface is sealed with a cobblestone pavement, which is mostly overgrown by grass. Below this anthropogenic surface, sandy gravels form the aquifer to a depth of 20 m in the northern area and 10 m in the southern part, followed by a layer of fine sandy alluvium. Underneath this sandy layer, at a depth of around 35 m in the northern area and 13 m in the southern part, a northwards-dipping aquitard is encountered, which consists of silty clays. Figure 1 (left) presents a satellite image of the abandoned site and its surroundings. The river Glan is situated about 70 m to the north of the factory border, exhibiting a southeast-directed flow. Infiltrating water from the river Glan into the aquifer dictates the undisturbed southeast-directed groundwater flow. Additionally, the local groundwater flow is strongly influenced by the production well of the tannery, which is operated discontinuously with a pump rate of 30 L s^−1^ for several minutes each day. The depth of the groundwater is around 7 m, featuring natural fluctuations, and a very low hydraulic gradient (0.35–0.5‰). The porosity of the aquifer is approximately 20%, and the hydraulic conductivity was determined to vary between 2.0 × 10^−03^ and 1.0 × 10^−04^ m s^−1^.

The Cr contamination beneath the factory area is known since 1987, and the site was the focus of various investigations. Necessary production materials, e.g., the Cr(III)-containing tannic acid, were manufactured on site. This tannic acid was produced by boiling potassium dichromate (K_2_Cr_2_^6+^O_7_) and sulfuric acid (H_2_SO_4_), followed by subsequent reduction with the organic reducing agent treacle (molasses). Spilling, leakage, inappropriate disposal practices, and/or mismanagement led to underground contamination (Umweltbundesamt 2013). Earlier investigations, carried out on behalf of the EAA, clearly identified two Cr(VI)-containing hot spots in the underground (see Fig. 1): the first (HS1) is situated beneath the former, demolished reduction works building; the second (HS2) is located in the vicinity of the new reduction building in the northern part of the factory area (UTC 2012; Umweltbundesamt 2013).

At HS1, the Cr(VI) contamination is located above the groundwater table, i.e., the vadose zone, whereas at HS2, it is situated in the groundwater-fluctuation zone at a depth between 6 and 8 m (Umweltbundesamt 2013). Sedlazeck et al. (2017) provide a detailed mineralogical and geochemical characterization of the contamination at HS2. In their description, they identified the source of the contamination, an old soakaway, from where the contaminated material (mostly liquids) infiltrated the alluvial deposits and subsequently the aquifer. As described above, a new approach for the remediation of Cr(VI) hot spots has been invented (Müller et al. 2014). These authors performed preliminary stress tests for the groundwater remediation and also examined the suitability of the applied chemicals. Subsequently, a pilot plant was installed near HS1, and first field tests were conducted, revealing promising results (Müller et al. 2014). The current paper focuses on the impact of the in-situ remediation on the groundwater during remediation work at HS1 and HS2 and reports the latest results.

### Remediation method

The remediation strategy (for details, see Müller et al. (2014)) consists of the manipulation of the redox potential of a system to reduce Cr(VI) to Cr(III) as described by the half-reaction

1 $$ {\mathrm{Cr}}_2{{\mathrm{O}}_7}^{2-}+14{\mathrm{H}}^{+}+{6\mathrm{e}}^{-}<=>{2\mathrm{Cr}}^{3+}+{7\mathrm{H}}_2\mathrm{O},{\mathrm{E}}_0=+1.33\ \mathrm{V}, $$

which requires electrons and is favored by acidic conditions (Park et al. 2004; Zheng et al. 2014; Aranda-García and Cristiani-Urbina 2019). To achieve this reduction in the field, a combination of an in-situ remediation method with a unique pump and treat technology is used. This combination leads to a reduction of Cr(VI) and an in-situ geochemical fixation of Cr(III) in contaminated soils through reduction-induced precipitation, and simultaneously, Cr(VI) is removed from the groundwater and treated ex-situ within the pump and treat unit. The resulting mixed Cr/Fe sludge (from the groundwater treatment, see below) is subsequently removed via a lamella clarifier and a chamber filter press (Müller et al. 2014).

For the in-situ treatment of the soil, sodium dithionite (Na_2_S_2_O_4_), a strong reducing agent, is dissolved in water (~ 6.7 g L^−1^ at HS1; ~ 24 g L^−1^ at HS2) and released in the contaminated area. The assumption is that the released ions -and/or their reaction products- will interact with available Cr(VI) to produce Cr(III) (Dresel et al. 2011). Sodium dithionite is a salt of the dithionous acid (H_2_S_2_O_4_) and is considered a weak base. The Na_2_S_2_O_4_ dissolution process produces the dithionite ion (S_2_O_4_^2−^), but this newly formed ion is rather unstable. Consequently, it will further react, generating to a variety of reaction products, as summarized by Amonette et al. (1994): dissociation and disproportionation produces sulfoxyl radicals (SO_2_^•-^), thiosulfate (S_2_O_3_^2−^), sulfite (SO_3_^2−^), and/or hydrogen sulfite (= bisulfite, HSO_3_^−^) (Mayhew 1978; Amonette et al. 1994; Su and Ludwig 2005; Saikhao et al. 2017). Even though redox reactions between S_2_O_3_^2−^, or its disproportionation products, and Cr are known (Avakian et al. 2005; Demoisson et al. 2007), these are not considered further because the species SO_2_^•-^, SO_3_^2−^, and HSO_3_^−^ are known to be the relevant ones for reducing Cr(VI) to Cr(III) (Palmer and Wittbrodt 1991; Amonette et al. 1994; Cheng et al. 2009; Kaprara et al. 2018). The standard potential for the production of HSO_3_^−^ from S_2_O_4_^2−^ (S_2_O_4_^2−^ + 2 H_2_O ⬄ 2HSO_3_^−^ + 2e^−^ + 2H^+^) is *E*_0_ = − 0.66 V at pH 7 and 25 °C, and the reaction will produce H^+^, leading to a decrease in pH in the solution (Mayhew 1978).

The dissociation of 1 mol Na_2_S_2_O_4_ produces 2 mol SO_2_^•-^, yielding a strong and very reactive reducing agent, which is able to directly reduce Cr(VI) to Cr(III). If ferric iron (Fe(III)) is present in solution or as structural Fe(III) in solids, the sulfoxyl-free radicals will readily reduce Fe(III) to ferrous iron (Fe(II)), which itself is able to directly reduce Cr(VI) to Cr(III), as seen in Eq. 2 (Mitrakas et al. 2011; Palmer and Wittbrodt 1991; Istok et al. 1999; Fruchter et al. 2000; Su and Ludwig 2005; Joe-Wong et al. 2017).

2 $$ \mathrm{Cr}{\left(\mathrm{VI}\right)}_{\left(\mathrm{aq}\right)}+3\mathrm{Fe}{\left(\mathrm{II}\right)}_{\left(\mathrm{aq},\mathrm{s}\right)}+{3\mathrm{e}}^{-}<=>\mathrm{Cr}{\left(\mathrm{II}\mathrm{I}\right)}_{\left(\mathrm{aq},\mathrm{s}\right)}+3\mathrm{Fe}{\left(\mathrm{II}\mathrm{I}\right)}_{\left(\mathrm{aq},\mathrm{s}\right)} $$

In addition, sulfite species, such as bisulfite, are also able to reduce Cr(VI) to Cr(III), as shown representatively for bisulfite in conditions of excess (bi)sulfite

3 $$ {6\mathrm{H}}^{+}+{{2\mathrm{HCrO}}_4}^{-}+{{4\mathrm{HSO}}_3}^{-}=>{2\mathrm{Cr}}^{3+}+{{2\mathrm{SO}}_4}^{2-}+{\mathrm{S}}_2{{\mathrm{O}}_6}^{2-}+{6\mathrm{H}}_2\mathrm{O}, $$

or excess Cr(VI) (Palmer and Wittbrodt 1991).

4 $$ {5\mathrm{H}}^{+}+{{2\mathrm{HCrO}}_4}^{-}+{{3\mathrm{HSO}}_3}^{-}=>{2\mathrm{Cr}}^{3+}+{{3\mathrm{SO}}_4}^{2-}+{5\mathrm{H}}_2\mathrm{O}. $$

Analogous reactions can also be written for other sulfite species. The S_2_O_6_^2−^ (dithionate), produced in reaction 3, is able to reduce Fe(III) to Fe(II), which provides a possible explanation of the supporting effect of present Fe for the reduction of Cr(VI) (Li et al. 2017).

The Na_2_S_2_O_4_ is dissolved in water in a storage tank prior to injection. After this dissolution, produced radicals react rapidly with ions present in solution. As the solid chemical is not applied continuously due to a high risk of ignition, the time between its dissolution and the injection of the solution is variable (to a maximum of 1 week); hence, it can be assumed that the radicals are not anymore present by the time of injection. Nevertheless, during injection, the reducing strength is still sufficient to reduce Cr(VI) to Cr(III).

Laboratory experiments during previous projects revealed that Cr(VI) can be mobilized due to ion exchange of the formed SO_4_^2−^ ions with adsorbed CrO_4_^2−^ ions at the soil mineral surfaces (Amonette et al. 1994; Müller et al. 2014). For the case that the reducing strength of the injected Na_2_S_2_O_4_ solution and the resulting (bi)sulfite and Fe(II) might not be high enough to instantly reduce the mobilized Cr(VI) quantitatively, the pump and treat groundwater decontamination system is installed to clean the groundwater. Consequently, further spreading of the harmful Cr(VI) is prevented. The groundwater is extracted by a well and is pumped through a cascade of reactors. These reactors are filled with Fe(0) granules (diameter approximately 5 mm), and the water flow (from bottom to top) induces a fluidization of the Fe(0) granule bed. Dissolved Cr(VI) is either directly reduced through the Fe(0)_(s)_ (Eq. 5) or Fe(II) (Eq. 2) that formed within the reactors through dissolution (and oxidation) of Fe(0)_(s)_.

5 $$ {{\mathrm{Fe}}^0}_{\left(\mathrm{s}\right)}+\mathrm{Cr}{\left(\mathrm{VI}\right)}_{\left(\mathrm{aq}\right)}+{3\mathrm{e}}^{-}<=>\mathrm{Cr}{\left(\mathrm{III}\right)}_{\left(\mathrm{aq},\mathrm{s}\right)}+\mathrm{Fe}{\left(\mathrm{III}\right)}_{\left(\mathrm{aq},\mathrm{s}\right)} $$

Reaction products of Eqs. 2 and 5 will precipitate according to

6 $$ \mathrm{XCr}{\left(\mathrm{III}\right)}_{\left(\mathrm{aq}\right)}+\left(1-\mathrm{X}\right)\mathrm{Fe}{\left(\mathrm{III}\right)}_{\left(\mathrm{aq}\right)}+{3\mathrm{H}}_2\mathrm{O}<=>{\mathrm{Cr}}_{\mathrm{X}}{\mathrm{Fe}}_{1-\mathrm{X}}{\left(\mathrm{OH}\right)}_{3\left(\mathrm{s}\right)}+{3\mathrm{H}}^{+}. $$

Sass and Rai (1987) discovered that the Cr-Fe hydroxide (Cr_X_Fe_1-X_(OH)_3(s)_), produced in Eq. 6, behaves thermodynamically like a solid solution and that the solubility of this solid solution is lower if high Fe concentrations are present.

By using Fe(0) within the reactors, constant availability of Fe(0) surface area is guaranteed by refilling the reactant itself and by abrasion of the passivated (oxidized) surface, yielding a practically unlimited amount of Fe(0) and, consequently, Fe(II) within the reactors, which are readily removing Cr(VI) from the system through reduction (Eqs. 2 and 5) and precipitation of the Cr-Fe hydroxide (Eq. 6). Additionally, the surface of this (amorphous) hydroxide phase acts as a newly formed sorbent for further fixation of Cr(VI) from the solution, as described, e.g., by Aoki and Munemori (1982) or Owlad et al. (2009).

Both processes, reduction-induced precipitation as well as sorption lead to the fixation of contaminants, and the produced solid particles can subsequently be removed from the system. Part of the remediated groundwater is mixed with Na_2_S_2_O_4_ and reinjected into the underground, whereas the rest is released into a nearby outlet channel, which drains into the river Glan (see Fig. 1).

During the course of this research project, two main injection strategies with little variation were applied. At HS1, where the contamination is located in the unsaturated zone, the reducing agent is injected at a depth of around 1 m by using injection lances, which were installed above the contamination (see Fig. 1, top right). The injected water percolates the subsurface, including the contamination, by a (mostly) gravity-driven flow until it infiltrates the groundwater. At HS1, 8 injection lances were used for the first 208 days. Subsequently, the injection grid was refined to 17 lances on the same base area to improve the dispersion of the released reducing agent, but the discharge rate was not increased. The injection was performed for another 32 days, adding up to a total of 240 days. At HS2, the contamination was located in the groundwater fluctuation zone. The reducing agent was released through an old soakaway, which was identified as the contamination source (see Fig. 1, bottom right). By injecting the reducing agent into the soakaway, it is ensured that it takes the same pathway as the contaminant did in the past. In addition to that, three groundwater wells were drilled upgradient of the contamination. After a certain period of time (see “Hot spot 2” section), the reducing agent was released via these three wells directly into the groundwater-driven flow that passes the contaminated area. A mixture of these injection strategies (2 injection wells and the soakaway) was also tested. At both hot spots, the extraction well for the remediated groundwater was located downgradient of the contamination. One goal of the project was to identify influences of the injection variations on the Cr(VI) removal. In total, around 4000 and 3450 kg of dissolved Na_2_S_2_O_4_ were released at HS1 and HS2, respectively.

### Groundwater monitoring

In order to observe the progress of the remediation, groundwater samples were taken on a 2-week basis according to (ÖNORM S 2092 2008). Some parameters, i.e., Eh (DIN 38404–6 1984), pH (ISO 10523 2012), electric conductivity (DIN EN 27888 1993), and groundwater level, were determined on-site. The redox potential was measured by using an Ag/AgCl 3 M KCl electrode, and the measured data were subsequently corrected to the standard hydrogen electrode. As redox measurements in waters are very problematic (Hostettler 1984; Peiffer 2000), the redox potential (Eh) is used here only to determine whether a system is more or less oxidizing. Among others, the pH strongly influences the redox conditions of a certain system. To eliminate this impact, the measured Eh values were corrected to pH 7 (Eh7), calculated according to: Eh7 = Eh−2.303 × R × T × F^−1^ × (7–pH), where *R* = 8.314 J mol^−1^ K^−1^ (ideal gas constant), *F* = 96,484.56 C mol^−1^ (Faraday constant), and *T* is the Kelvin temperature.

Samples were conserved according to (ISO 5667-3 2012) in high-density polyethylene (HDPE) bottles and sent to the Montanuniversitaet Leoben where the concentrations of Cr_(tot)_, Cr(VI), Fe_(tot)_, Fe(II), SO_3_^2−^, and SO_4_^2−^ were determined in every sample in the laboratory of the Chair of Waste Processing Technology and Waste Management.

The Cr_(tot)_ and Fe_(tot)_ concentrations were measured according to DIN EN ISO 17294-2 (2005) by using an inductively coupled plasma mass spectrometer (ICP-MS; Agilent Type 7500). Sulfite, Cr(VI), and Fe(II) concentrations were determined photometrically with an Unicam UV4 spectrometer, following Pachmayr (1960), DIN 38405–24 (2003), and DIN 38406–1 (1983). Sulfate concentrations were measured via ion chromatography according to DIN EN ISO 10304-1 (2009) by using a DIONEX-IC (ICS 2000).

## Results and discussion

### Hot spot 1

#### Cr(VI) removal (day 0 to day 240)

**Fig. 2:**
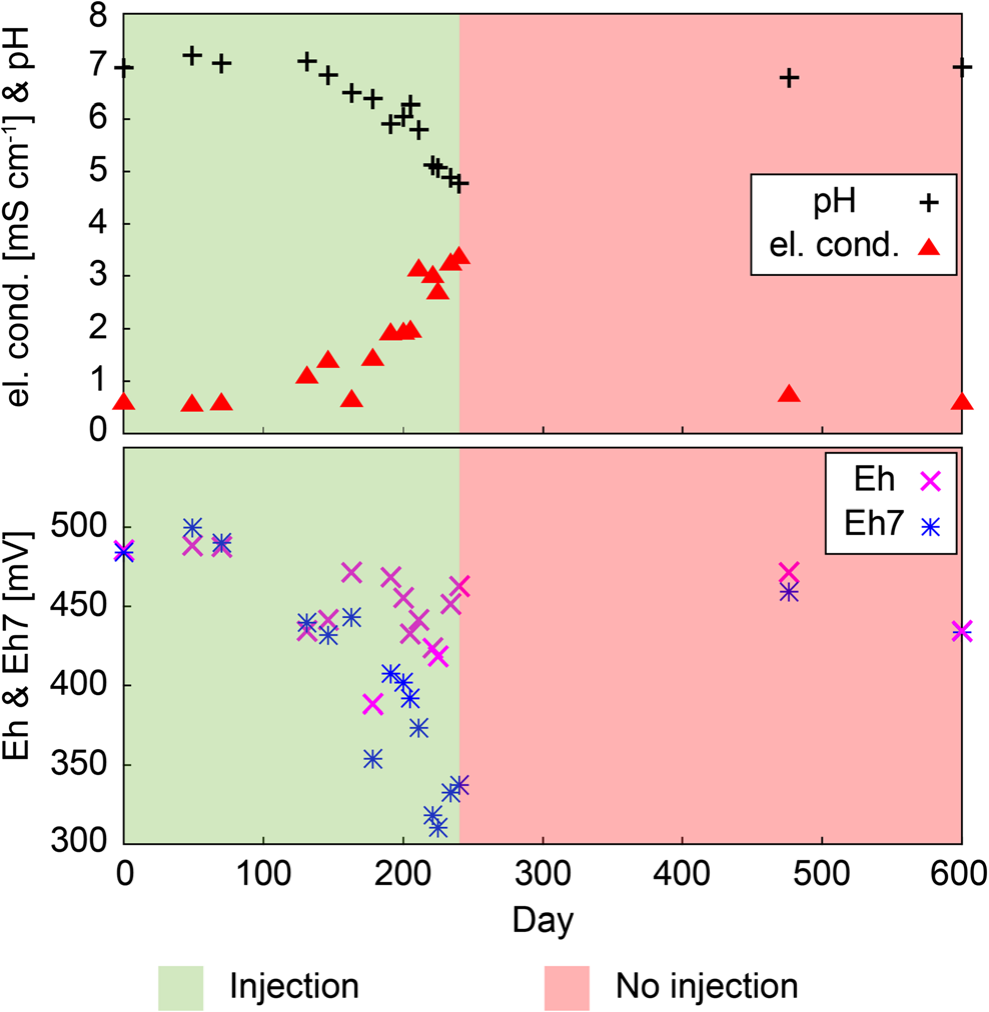
Electric conductivity, pH, Eh, and Eh7 of groundwater samples taken throughout the remediation project from the well at HS1

**Fig. 3:**
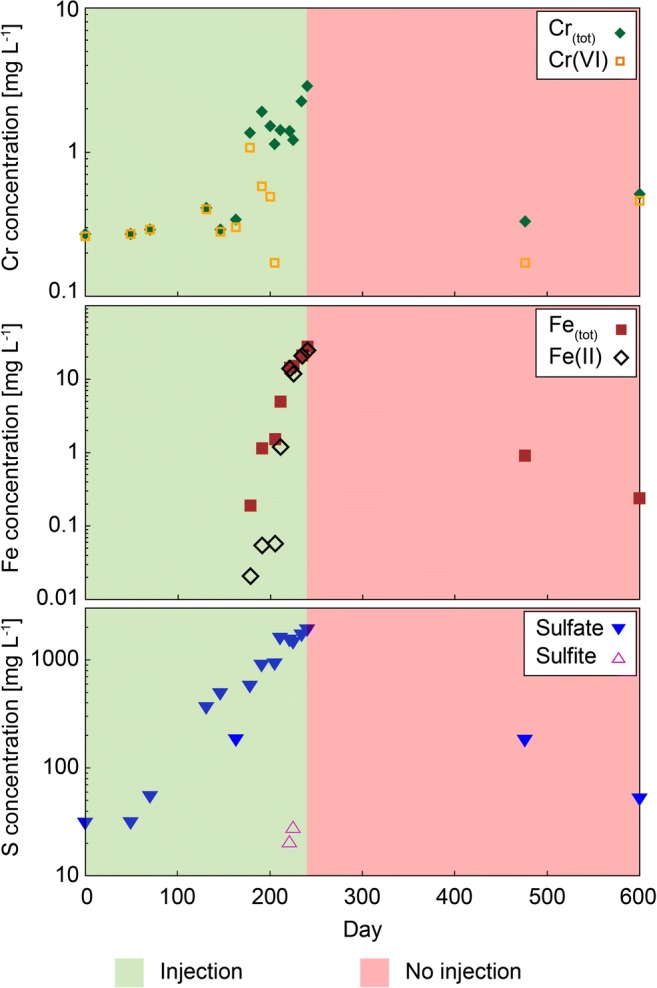
Concentrations of Cr_(tot)_, Cr(VI), Fe_(tot)_, Fe(II), sulfate, and sulfite in groundwater samples taken throughout the remediation project from the well at HS1. If concentrations were below the limit of detection, no data point is plotted

In 2014, the field work focused on the decontamination of hot spot 1 (HS1). Measured on-site parameters are presented in Fig. 2, the results of measured ion concentrations in Fig. 3. The baseline measurement for the original groundwater before injection of Na_2_S_2_O_4_ corresponds to day 0 in all diagrams. One day after the baseline measurement, injection of the reducing agent started. Due to technical problems, no sampling was possible between days 70 and 130. The 2014 field campaign ended on day 240.

On-site measurements of the original conditions revealed a neutral pH of 7.0, an Eh of 486 mV, and an electric conductivity of 0.57 mS cm^−1^. Due to the lack of data (days 70–130), the exact arrival of the reducing agent at the groundwater cannot be ascertained. However, starting on day 131, the electric conductivity increased, whereas pH decreased, suggesting that the reducing agent had arrived in the groundwater (Fig. 2). The injection did not show a definite influence on Eh, but Eh7 displays a marked drop beginning on day 131.

An almost steady increase of the Cr_(tot)_ concentration until day 240 has been observed (Fig. 3). Until day 178, most of the Cr_(tot)_ was present as Cr(VI), which exhibited its maximum concentration of 1.07 mg L^−1^ on day 178. Subsequently, the Cr(VI) concentration decreased. Increasing sulfate concentrations have been observed between days 70 and 240. Sulfite has only been detected on two sampling days, when calculated Eh7 values were very low and shortly after Cr(VI) was no longer detected in the groundwater.

Prior to the chromate peak on day 178, both Fe_(tot)_ and Fe(II) concentrations were below their detection limits of <0.004 and <0.02 mg L^−1^, respectively (Fig. 3). After day 178, the concentrations of Fe_(tot)_ as well as Fe(II) almost continuously increased until the end of the 2014 field campaign (day 240). After day 205, when Cr(VI) was no longer detected in the groundwater, the proportion of Fe(II) to Fe_(tot)_ increased significantly, and almost all Fe_(tot)_ was present as Fe(II).

Between the arrival of the reducing agent and day 178, the situation can be described by Eq. 4, i.e., by the presence of excess Cr(VI). Part of the mobilized hexavalent Cr is constantly reduced by sulfite (and probably Fe(II)) and subsequently removed by precipitation of the Cr-Fe hydroxide (Cr_X_Fe_1-X_(OH)_3_). Sulfite is completely consumed, leading to an increase of the sulfate concentration. Produced sulfate ions enhance the described desorption of CrO_4_^2−^ ions from the surfaces of soil minerals (Amonette et al. 1994; Müller et al. 2014). Between day 178 and the appearance of sulfite in the groundwater (day 221), the reaction conditions gradually changed to those, described by Eq. 3, because the Cr(VI) concentration decreased through reduction by sulfite, which is continuously provided through the injection, but Eq. 3 is not completely satisfied yet. Associated with these changing conditions to Eq. 3, S_2_O_6_^2−^ is already produced, which reacts with Fe(III) to Fe(II). Lesser amounts of oxidation partners, i.e., Cr(VI), are being present for further reaction, resulting in an increase of the Fe(II) concentration. Shortly after Cr(VI) is removed from the solution, sulfite was detected in the groundwater (days 221 and 225), suggesting that conditions, described by Eq. 3 (excess sulfite), are satisfied.

During the first 178 days, the absence of both Cr(III) and Fe(III) can be considered as proof for the precipitation of a Cr-Fe hydroxide according to Eq. 6. The described Cr(VI) mobilization (desorption) and subsequent reduction after day 178 leads to an increase in the Cr_(tot)_ concentration in the groundwater. However, the removal of Cr_(tot)_ through precipitation of the Cr-Fe hydroxide was prohibited due to instant reduction of (dissolved and newly formed structural) Fe(III) to Fe(II) by the injected reducing agent (Sass and Rai 1987; Palmer and Puls 1994; Su and Ludwig 2005; Saikhao et al. 2017). Except for the end of the injection period (after day 211), when the pH was relatively low (pH < 6), the pH-controlled dissolution of Cr(III) hydroxide, or Cr(III)-Ca-containing hydrocalcite, which were inferred to be present at this site by Sedlazeck et al. (2017), can be excluded as an additional source for increasing Cr_(tot)_ (i.e., Cr(III)) concentrations.

#### Rebound effect (day 240 to day 600)

In the following year (2015), on days 476 and 600, i.e., 236 and 360 days after terminating the injection, two additional groundwater samples were taken from the same groundwater well at HS1. Figure 2 shows that the three on-site parameters converged almost to the original values determined on day 0.

Figure 3 documents that the Cr_(tot)_ concentration decreased again, but that nearly half of it was present as Cr(VI). On day 600, almost 1 year after the injection was terminated, concentrations of Cr_(tot)_ and Cr(VI) even exceeded the concentrations on day 0, but they are still in the range described by previous observations for this site (Umweltbundesamt 2013). The sulfate and Fe_(tot)_ concentrations were elevated, but lower than at the termination of the injection; neither sulfite nor Fe(II) were detected because they were consumed for chemical reactions with Cr(VI) and/or washed out by the natural water flow.

The system’s redox conditions on day 600 were more reducing than those determined at the beginning of the remediation experiment. However, the redox potential of the system was not sufficiently low to reduce the freshly dissolved/desorbed Cr(VI). This feature of reappearance of the contaminant, the rebound effect, is known from the literature for various contaminants (Cohen et al. 1994; Adamson et al. 2011; Held 2014), but it has not been described for Cr(VI).

### Hot spot 2

**Fig. 4:**
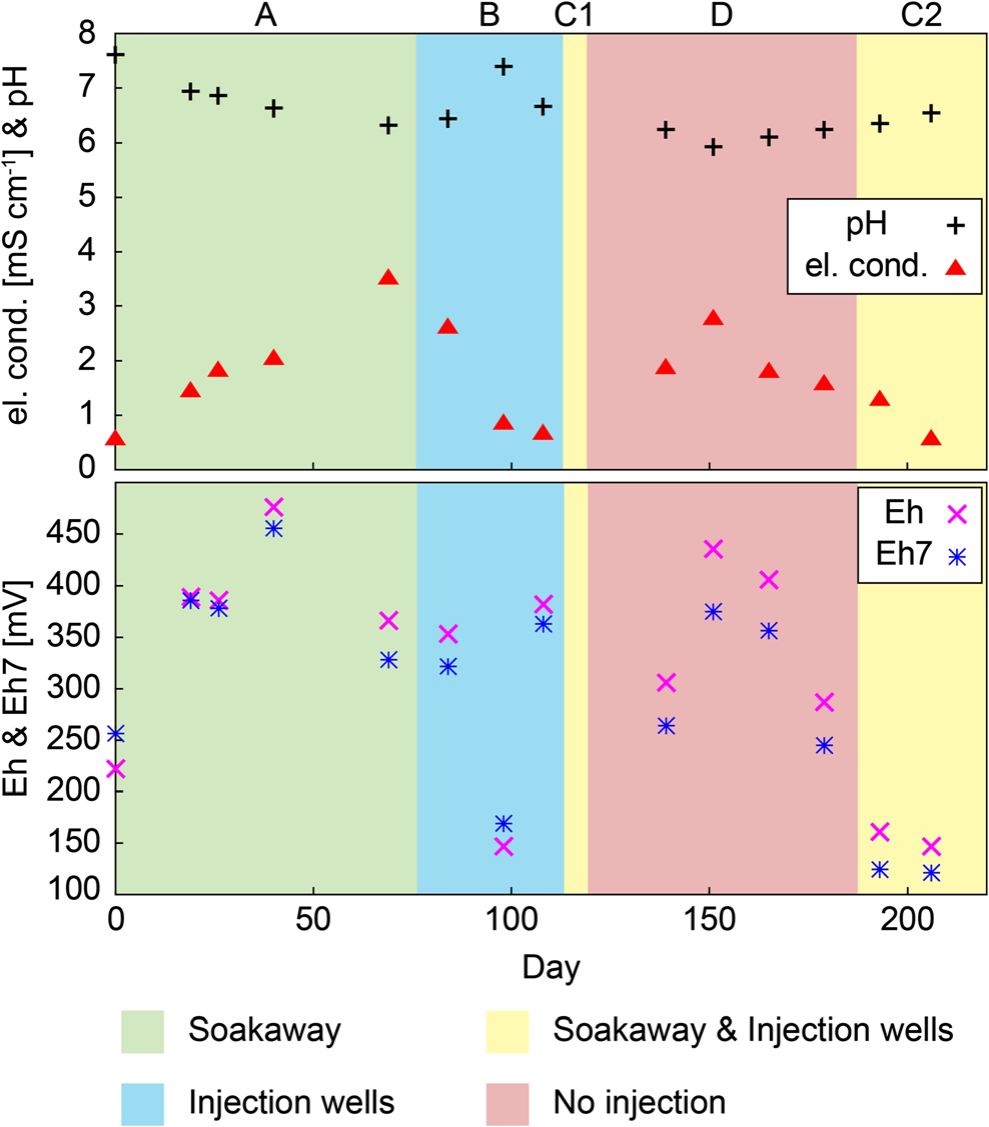
Electric conductivity, pH, Eh, and Eh7 of groundwater samples taken throughout the remediation project from the well at HS2. Background colors and letters A–D represent different injection pathways

**Fig. 5:**
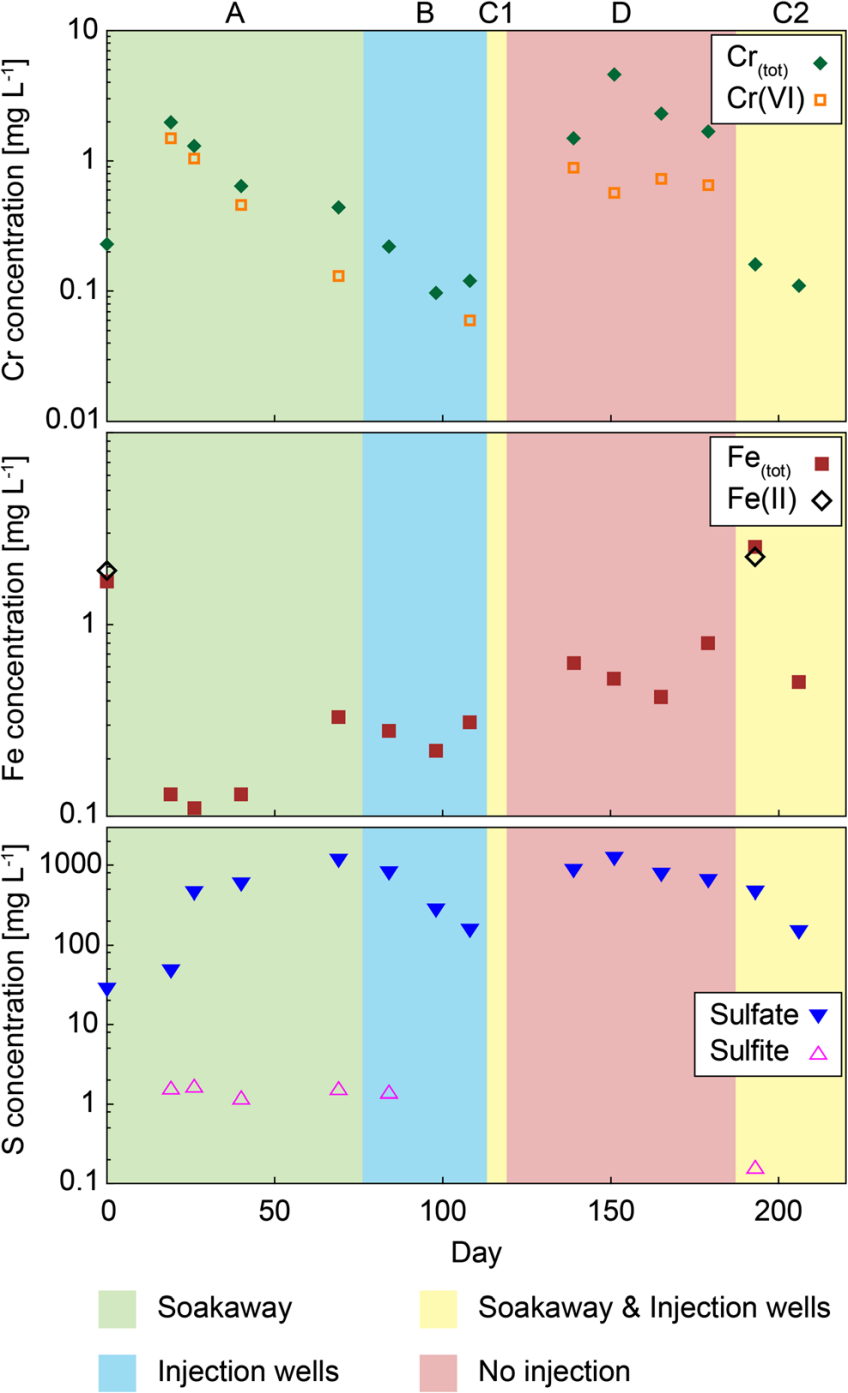
Concentrations of Cr_(tot)_, Cr(VI), Fe_(tot)_, Fe(II), sulfate, and sulfite in groundwater samples taken throughout the remediation project from the well at HS2. Background colors and letters A–D represent the chosen injection pathways. If concentrations were below the limit of detection, no data point is plotted

In contrast to HS1, where the reducing agent was only released through the injection lances, different injection pathways were chosen at hot spot 2 (HS2), as shown by different background colors and the letters A–D in Figs. 4 and 5. Field work at HS2 was carried out in 2015.

Between days 1 and 76 (A, green), the reducing agent was directly released into the old soakaway, which has been identified previously as the source of the Cr(VI) contamination (Sedlazeck et al. 2017). During this period, no other injection pathway was used, and the reducing agent was released at a depth of 4 m. From day 76 until day 113, three injection wells, upgradient of the contamination (see Fig. 1, bottom right), were chosen for direct injection into the groundwater (B, blue). A technical error forced interruption of the injection (days 119–187), shown in red (D). Prior to this interruption (days 113–119), a combination of two injection wells and the soakaway (C1, yellow) was chosen. After the no-injection period, the Na_2_S_2_O_4_ solution was released again through the soakaway and two injection wells (C2, yellow) until the end of the project (day 206).

The baseline measurements on day 0 revealed that at HS2, the electric conductivity (0.56 mS cm^−1^) was similar to that determined at HS1, but the conditions were more alkaline (pH = 7.6) and more reducing (222 mV). After starting the injection via the soakaway, the pH dropped and the electric conductivity started to increase (Fig. 4). An increase of the redox potential was determined during the first few weeks, followed by a sharp drop on day 69. After moving the injection from the soakaway to the injection wells (blue, B), pH, and electric conductivity returned to values near the baseline, whereas the redox potential fluctuated considerably.

Prior to the no-injection period, the reducing agent was injected through two injection wells and the soakaway (C1). This short injection period and the previous injection period (B) were sufficient to raise the electric conductivity as well as the redox potential during the period without injection (red, D), which was accompanied by a pH drop to the lowest level measured during this campaign (day 151). Subsequently, the pH became more alkaline again, whereas both Eh and the electric conductivity dropped continuously until the end of the field work.

The behavior of the sulfate concentration (Fig. 5) in the groundwater was similar to that of the electric conductivity (Fig. 4). Sulfite was present during the first injection period (soakaway) and in the samples taken on days 84 and 193. High Cr_(tot)_ and Cr(VI) concentrations were detected during periods A and D, with Cr mostly present as Cr(VI) during the injection via the old soakaway (A), and a smaller proportion of Cr(VI) during the period of no injection (D). Hexavalent Cr was also present on day 108, at the end of the second injection period (B). After starting the Na_2_S_2_O_4_ injection, the concentrations of both Fe_(tot)_ and Fe(II) decreased. Over time, however, the Fe_(tot)_ concentration rebounded, except for the last sample (day 206). Fe(II) was only detected in two samples (days 0 and 193).

After starting the injection, Cr(VI) is mobilized, and the original reducing conditions changed to the excess sulfite conditions as described by Eq. 3. Produced S_2_O_6_^2−^ reacts with present Fe(III) to form Fe(II), which then initiated the reduction-induced precipitation of the Cr-Fe hydroxide (Eqs. 2 and 6), leading to a continuous decrease of the Cr concentration in the groundwater until day 98. Nevertheless, direct reduction of Cr(VI) by sulfite and/or the sulfoxyl free radicals is not sufficient because sulfite and Cr(VI) occurred together, suggesting that the presence of Fe during the Cr reduction is the rate-limiting step in the reduction process. Consequently, we assume that the reduction rate would increase if more Fe was present in the groundwater. After moving the injection to the groundwater wells upgradient of the contamination (day 84), the pH increased and the electric conductivity decreased to the level of the baseline measurement, suggesting that the already injected reducing agent was washed out, and the newly injected reducing agent did not yet reach the extraction well. This is also supported by the decreasing sulfate and Cr_(tot)_ concentrations and the disappearance of sulfite (days 98 and 108). On day 108, Cr(VI) was detected, and the redox potential was elevated, which might result from a rise of the groundwater level (Fig. S1, appendix), leading to the desorption of Cr(VI) or to the dissolution of Cr(VI)-bearing solid phases, which were inferred to be present in the groundwater fluctuation zone (Sedlazeck et al. 2017). At this point, neither sulfite nor Fe(II) were present to reduce the mobilized Cr(VI).

An additional change of the injection method was used for 6 days (C1, yellow), prior to period D, during which no reducing agent was injected. Consequently, high Cr(VI) abundances were measured. However, not the entire amount of Cr_(tot)_ was present as Cr(VI), suggesting that a certain amount of the mobilized Cr(VI) was being reduced. Nevertheless, if Fe(II) and sulfite were present, they have instantly been completely used up for the reduction of Cr(VI). The short injection period (C1) was not sufficient to change the conditions to excess sulfite (Eq. 3), and low Fe(III) concentrations can be considered as a proof for the precipitation of the Cr-Fe hydroxide (Eq. 6), although not the entire amount of Cr(III) was removed by this precipitation reaction.

After restarting the injection on day 187, sulfite is present again, and no Cr(VI) was detected (day 193) as described by the excess sulfite reaction provided in Eq. 3 and the produced S_2_O_6_^2−^ reacted with Fe(III) to Fe(II) which was also present in solution.

### Comparison of the two hot spots

The initial Cr_(tot)_ concentration in the groundwater at HS1 was low (0.27 mg L^−1^), but most of the Cr was present as Cr(VI), hence, the concentration exceeded the threshold value for Cr(VI) in groundwater (0.01 mg L^−1^; ÖNORM S 2088–1 2004) by almost 30 times. At HS2, similar low Cr_(tot)_ concentrations were observed (0.23 mg L^−1^), but no Cr(VI) was detected on day 0. These results are consistent with the measured redox conditions as the system at HS1 was more oxidizing (Eh7 = 500 mV) compared with HS2 (Eh7 = 260 mV).

The increase of the redox potential to the approximate starting conditions of HS1, after starting the injection at HS2 probably results from the mobilization of oxidized aqueous species, i.e., chromate. The interaction of the injected ions with solid phases in the subsurface leads to an additional mobilization of Cr(VI) (Müller et al. 2014), which may be present in the alluvial sediments either as discrete Cr(VI)-bearing solid phases above the groundwater level (vadose- and groundwater-fluctuation zone; Sedlazeck et al. (2017)) or as adsorbed species as suggested by Amonette et al. (1994). This mobilization is most likely represented by the Cr(VI) peaks on day 178 at HS1 and on day 19 but also by the high concentration on day 139 at HS2. The arrival of the Cr(VI) peak at HS2 occurs much sooner after the start of the injection, which is probably due to the smaller distance between the injection point and the extraction well. This early appearance of the Cr(VI) peak could, however, also be due to the existence of predefined pathways in the underground, because the soakaway has been used over the years for discharging contaminated waters from the factory into the aquifer. These pathways were used by the reducing agent as well, whereas at HS1, the injected reducing agent had to find a pathway through the underground, probably leading to a much higher diffusion and the formation of a reduction-front line, which leads to the drop of the redox potential.

At HS1, the abundance of Cr(VI) dropped soon after the detected maximum, while an increase of Cr_(tot)_ has been observed. At HS2, the Cr(VI) as well as Cr_(tot)_ concentration decreased with time, but the Cr(VI) removal took approximately twice as long as at HS1. These differences can be explained by the prevailing reaction conditions: at HS1, the conditions gradually changed from excess Cr(VI) to excess sulfite during the experiment, whereas at HS2, the conditions changed from excess sulfite to excess Cr(VI) and to excess sulfite again at the end of the field work.

The presence of Cr in the groundwater is controlled by the presence of Fe species, which on the other hand are controlled by dissolution and precipitation reactions of Fe-bearing minerals. These reactions are strongly influenced by changes in pH. At HS1, no Fe was detected at the beginning of the experiment, but with ongoing injection, Fe_(tot)_ and Fe(II) concentrations increased quickly (between days 178 and 240); the precipitation of a Cr-Fe hydroxide (Eq. 6) was precluded by instant reduction of (dissolved and newly formed structural) Fe(III) to Fe(II) (between days 178 and 240) and additionally by the acidic pH (between days 211 and 240). At HS2, Cr-Fe hydroxide was formed, thereby removing Fe and Cr slowly from the system, except for the no-injection period when not all of the Cr(III) was removed by this precipitation reaction. This conclusion is supported by the observed decrease of the Fe_(tot)_ concentration compared with the baseline measurement at HS2, and by the increase in Fe_(tot)_, i.e., Fe(II) concentration at HS1. The discrepancy in dissolved Fe concentrations between HS1 and HS2 may result from three different conditions: 1) more Fe-bearing minerals present at HS1, which is not likely because there is only a small distance between the two hot spots; 2) the pH is not low enough to dissolve more Fe-bearing mineral phases at HS2; and 3) the residence time of the injected reducing agent in the underground of HS2 is too short for efficient reaction with Fe(II)-containing minerals, such as, chlorite (White and Yee 1985; Eary and Rai 1989; Palmer and Wittbrodt 1991; Brigatti et al. 2000). This mineral is indeed present in the sediments at HS2 (Sedlazeck et al. 2017).

### Techno-economic and ecological evaluation

The application of the injection of sodium dithionite led to a temporary removal of Cr(VI) from the groundwater, but unfortunately, it was also accompanied by a considerable increase of the sulfate concentration in the groundwater. A high demand of the reductant sodium dithionite is caused by the consumption of this compound by competing reaction partners (e.g., dissolved oxygen and/or other oxidized species) present in the underground. From both an economic and an ecological point of view, this in situ treatment is not only expedient due to the high quantities of required reductant and resulting high-sulfate concentrations, but also because of the observed rebound of Cr(VI), and thus, it is not sustainable. Sodium dithionite has been applied successfully as a reductant for Cr(VI); however, it was applied ex situ to treat Cr(VI)-contaminated water (e.g., Kaprara et al. 2014). An ex situ pump-and-treat process has the advantage that less sodium dithionite is required compared to our in situ process because fewer reaction partners are present in a pumped water compared to the underground, which represents a mixture of soil solution, a gaseous phase, and solid constituents. Another advantage of an ex situ process would be that the occurrence of the observed rebound can be excluded. However, the soil solution cannot be extracted easily due to high capillary forces, and additionally, Cr(VI) is also present in solid mineral phases (Sedlazeck et al. 2017). Consequently, we aimed for the development of an in situ process to quantitatively reduce Cr(VI) to Cr(III), where no further water treatment would have been required. In summary, the investigated in situ process revealed various technical, economic, and ecological challenges, but ex situ pump-and-treat processes can only treat groundwater and not the underground. Consequently, novel technologies need to be developed for a sustainable in situ reduction of Cr(VI).

## Conclusions

The experiments at both hot spots clearly show that Cr(VI) can be effectively removed from the system, at least temporarily, by injected sodium dithionite, and also by Fe(II), which is produced through reduction by S_2_O_6_^2−^ and through interaction with Fe-bearing mineral phases. During the experiments, different remediation phases of excess chromate and excess sulfite were identified while varying the injection strategy. Furthermore, the experiments revealed that the presence of Fe(II) can be the rate-limiting step in reducing Cr(VI) to Cr(III). However, measurements at HS1 revealed a rebound in Cr(VI) after terminating the injection. The observed rebound shows that the time period of injection was not long enough for either a quantitative reduction of Cr(VI) to Cr(III) and/or a total mobilization of present Cr(VI). Additionally, the dithionite concentration might not have been high enough. The mobilization of the Cr(VI) through the injection of Na_2_S_2_O_4_ explicitly shows the significance of a groundwater treatment to prevent a further dissemination of the contaminant.

## Electronic supplementary material

ESM 1 (DOCX 49 kb)
