# Supplementary material for: Impact of an in-situ Cr(VI)-contaminated site remediation on the groundwater
Source: Environ Sci Pollut Res Int. 2020 Jan 14;27(13):14465–75. doi: 10.1007/s11356-019-07513-9 (PMC7190687; doi:10.1007/s11356-019-07513-9)
Supplement: Supplementary file 1 — (DOCX 49 kb) [file 11356_2019_7513_MOESM1_ESM.docx]

# Supporting material


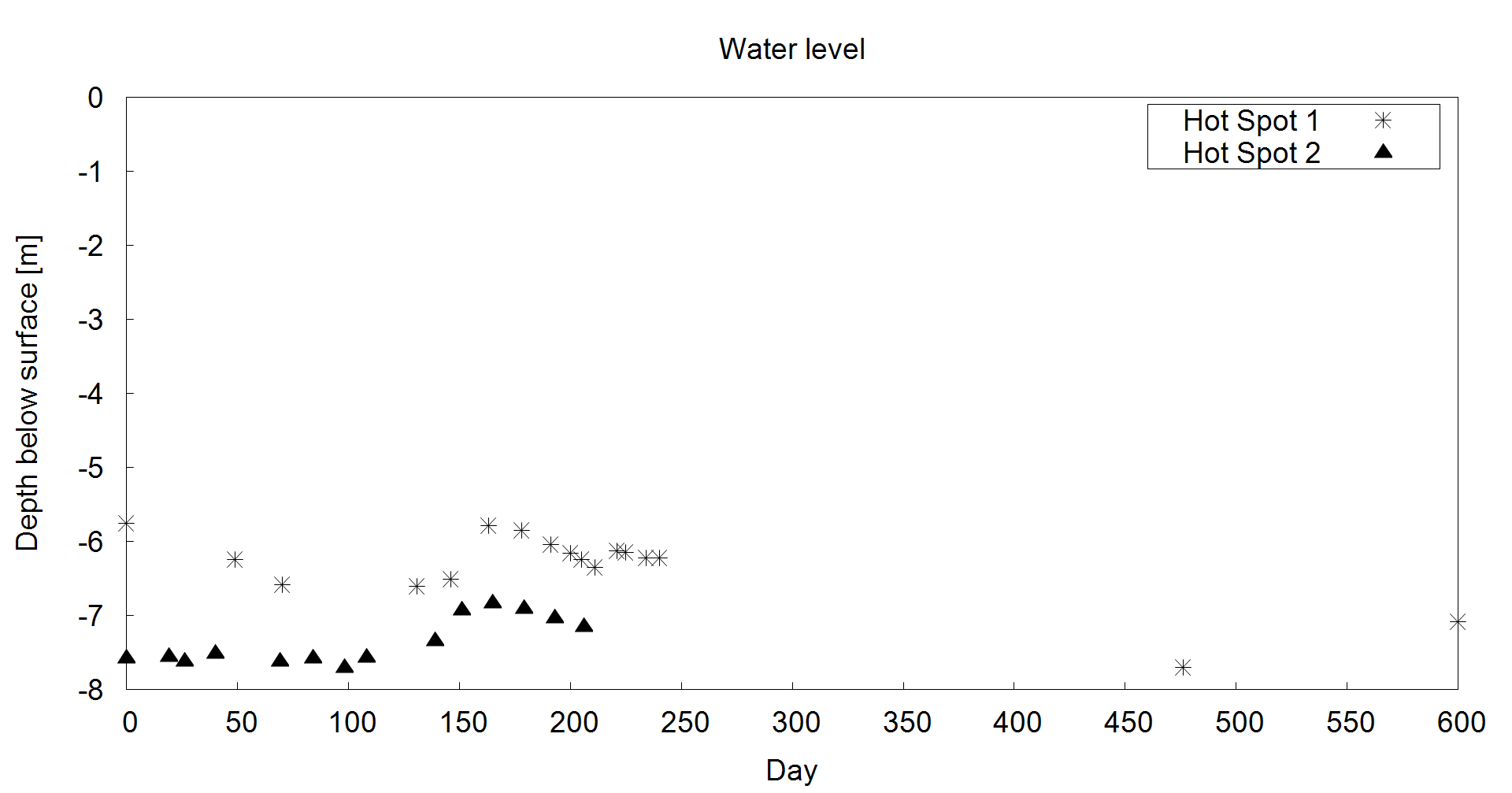
Figure S1: Water level below the surface throughout the remediation project from the wells at HS1 (2014 & 2015) and HS2 (2015). Day 0 corresponds to the first measurement, taken from the respective hot spot.
